# Clinical value of ^18^F-FDG PET/CT to predict MYCN gene, chromosome 1p36 and 11q status in pediatric neuroblastoma and ganglioneuroblastoma

**DOI:** 10.3389/fonc.2023.1099290

**Published:** 2023-03-24

**Authors:** Jiazhong Ren, Zheng Fu, Yaqing Zhao

**Affiliations:** ^1^ Department of Medical Imaging, PET-CT Center, Shandong Cancer Hospital and Institute, Shandong First Medical University and Shandong Academy of Medical Sciences, Jinan, Shandong, China; ^2^ Department of General Affairs Section, The Second Affiliated Hospital of Shandong University of Traditional Chinese Medicine, Jinan, Shandong, China

**Keywords:** neuroblastoma, ganglioneuroblastoma, ^18^F-FDG PET/CT, MYCN, 1p36, 11q

## Abstract

**Objective:**

To explore the value of ^18^F-2-fluoro-2-deoxyglucose (FDG) positron emission tomography (PET)/computed tomography(CT) in MYCN gene and chromosome 1p36 and 11 statuses in newly diagnosed pediatric NB(neuroblastoma) and GNB(ganglioneuroblastoma).

**Methods:**

We retrospectively analyzed newly diagnosed patients with 48 NB and 12 with GNB in our hospital. The data obtained from the clinical medical records included age, sex, pathologic type, and laboratory parameters such as lactate dehydrogenase (LDH), neuron-specific enolase (NSE) and the status of MYCN gene and chromosome 1p36 and 11q. The bone conditions were also obtained in the examination of bone marrow biopsy. Primary tumors were manually segmented to measure the maximum standardized uptake value (SUVmax), mean standardized uptake value (SUVmean), tumor volume(MTV) and total lesion glycolysis(TLG) and the maximal length of the lesion in the axial image(LEGmax).

**Results:**

The differences in bone marrow involvement and lymph node metastases in patients with chromosome 11q deletions were statistically significant (all p < 0.05). Chromosome 11q deletion was an independent factor affecting bone marrow involvement (OR=17.796, p=0.011). The levels of NSE, LDH, LEGmax and SUVmax, SUVmean, MTV, TLG all predicted MYCN gene amplification (all p < 0.05). The levels of LDH, LEGmax and MTV, TLG all predicted deletions in chromosomes 1p36 (all p < 0.05), while NSE, SUVmax and SUVmean did not (all p > 005).

**Conclusion:**

The LDH levels, LEGmax, MTV and TLG can effectively predict the status of the MYCN oncogene and chromosome 1p36 in pediatric neuroblastoma and ganglioneuroblastoma. Those patients with chromosome 11q deletions are more likely to develop bone marrow involvement and lymph node metastases, showing a worse progression-free survival.

## 1 Introduction

Neuroblastoma is the most common pediatric solid tumor in the sympathetic nervous system and accounts for approximately 15% of childhood cancer‐related mortality (1). Seventy percent of patients with NB have metastatic disease. The time of diagnosis, which commonly involves the cortical bone and the bone marrow, implies a poor prognosis (2, 3). Several clinicohistopathological factors, including age, stage (distant metastases in lymph nodes, cortical bone, bone marrow, and liver), laboratory test parameters (neuron-specific enolase (NSE), lactic dehydrogenase (LDH)) and molecular pathology (the status of MYCN gene, chromosome1p36 and 11q) (4, 5).

Some authors suggest that any gene and chromosomal segmental alteration is associated with an increased risk of relapse and poor outcomes in neuroblastoma[6]. Currently, the most clinically useful genetic marker is an amplification of the MYCN oncogene (6). In approximately 25% of primary untreated patients amplification of the MYCN oncogene is frequently associated with a poor outcome (6). However, specific segmental chromosomal aberrations, such as 11q deletion,1p deletion and 17q gain, are also biological factors associated with poor prognosis (5). The 2-year event-free survival rate for 11q deletion cases was 30%, compared to 34% for MYCN-amplified patients and 100% for cases without these abnormalities (6). Helena Carén et al. revealed that the MYCN-amplified and 11q-deletion groups are significantly associated with poor prognosis (7). Deletion of the short arm of chromosome 1p36 is one of the most characteristic changes in neuroblastomas (8), suggesting that a tumor suppressor gene resides in this region (9). A strong correlation between 1p36 deletion, MYCN amplification, and advanced-stage disease (10). Chromosome 17q gain is observed in approximately half of all tumors and is associated with a low survival rate (11).

Nowadays, iodine-123 metaiodobenzylguanidine (^123^I-MIBG) scintigraphy is a mainstay method in pediatric NB (12). Nevertheless, ^123^I-MIBG scintigraphy imaging has several disadvantages, such as no concentration of MIBG in 10% of tumors, limited spatial resolution, and limited sensitivity in small lesions. ^18^F-fluorodeoxyglucose (^18^F-FDG) positron emission tomography (PET)/computed tomography (CT) (^18^F-FDG PET/CT) is commonly used to complete the staging and prognosis prediction of neuroblastoma. Compared with ^123^I-MIBG scintigraphy imaging, superiorities of PET are high ^18^F-FDG avidity of the bone marrow and better identification of FDG abnormalities in the bone marrow and bone (13, 14). Moreover, another critical advantage of PET/CT is that PET/CT can assess the whole body at once and find unintended distant lymph nodes, bone and skin metastases where traditional imaging or bone marrow biopsies are not usually performed (15). ^18^F-FDG PET/CT has an excellent overall diagnostic accuracy with high sensitivity and specificity in detecting bone-bone marrow involvement and soft-tissue lesions in pediatric neuroblastoma (13, 16, 17). ^18^F-FDG PET/CT scans may help assess the full extent of disease involvement, particularly at therapeutic decision points (18).

^18^F-FDG PET/CT can be used for tumor characterization and prognostic assessment in patients with neuroblastoma (18). Tumor metabolic activity was higher in higher-stage MYCN-amplified patients (19). However, little research has studied the relationship between the status of the MYCN gene and 1p and 11q chromosomal and ^18^F-FDG PET/CT metabolic parameters. This study aimed to explore the correlation between ^18^F-FDG PET/CT metabolism parameters and the status of the MYCN gene, chromosomal 1p and 11q in newly diagnosed pediatric neuroblastoma by reviewing our clinical experience.

## 2 Materials and methods

### 2.1 Patients

This retrospective study was approved by the Institutional Review Board of the Affiliated Cancer Hospital of Shandong First Medical University and was exempted from obtaining informed consent. We retrospectively collected all the data of patients with neuroblastoma (age < 18 years) who had undergone ^18^F-FDG PET/CT before treatment from June 1, 2019, to August 31, 2022. Sixty patients were included, including 48 patients with NB and 12 with GNB. They were diagnosed for the first time and underwent ^18^F-FDG PET/CT scan. Some patients underwent bone marrow biopsy (BMB) within one week. Patients were undergoing any treatment procedures before ^18^F-FDG PET/CT were excluded.

The data obtained from the clinical medical records included age, sex, pathologic type, and laboratory parameters, such as lactate dehydrogenase (LDH), neuron-specific enolase (NSE), and the status of MYCN gene and 1p and 11q chromosome. All methods are performed according to the relevant guidelines and regulations. Clinical data was reviewed to assess disease status after follow-up. Progression-free survival (PFS) was calculated as the time from the ^18^F-FDG PET scan showing disease progression or if the patient died.

Pediatric neuroblastoma usually has three histological types: NB, GNB, and ganglioneuroma (GN). We excluded GN because it is a benign tumor (20) and not present with bone-bone marrow involvement. We used the international neuroblastoma risk group staging system (INRGSS) and the INRG risk classification system for neuroblastoma (21).

### 2.2 ^18^F-FDG PET-CT imaging protocol

^18^F-FDG is produced by a MiniTrace Cyclotron and automatic synthesis system by GE Healthcare, with a radiochemical purity of more than 95%. Patients fasted for at least six hours before the examination and had blood glucose lower than 10 mmol/L. The intravenous injection of FDG ranged from 4.44 MBq/kg to 5.55 MBq/kg. Thirty-eight patients were given oral sedation for PET scans. PET/CT scans were performed 60 minutes after injecting radiolabeled ^18^F-FDG using a Siemens PET/CT system (Horizon). The examination included a head-to-toe CT scan (80 kV; 50-100 mAs) and a three-dimensional (3D) PET scan (2 mins per bed; 6-7 beds). The rotation time was 0.6. The slice thickness was 3.75 mm. The increment was 3.27. The pitch was 0.984. The images were displayed on the Syngo.via workstation.

### 2.3 ^18^F-FDG PET/CT image analysis

Two experienced nuclear medicine physicians independently reviewed ^18^F-FDG PET/CT findings. Depending on the axis and the coronal and sagittal projections, the physicians placed the region of interest (ROI) on the primary tumor. Metabolic parameters, such as maximum standardized uptake value (SUVmax), average standard uptake value (SUVmean), metabolic tumor volume (MTV), and total lesion glycolysis (TLG), were measured on PET/CT images. TLG was then calculated as TLG = SUVmean × MTV. Our study selected a SUVmax of 30% as the threshold for generating the ROI. Meanwhile, the maximal length of the lesion in the reconstructed axial image(LEGmax) was also measured and collected in the CT features of the ^18^F-FDG PET/CT scan.

### 2.4 Identification of bone marrow disease by iliac crest biopsy

At our hospital, BMBs are obtained by pediatric oncologists from the common area of the posterior iliac epicondyle only and are not collected from other sites. Biopsy results are reported according to histopathology and immunophenotyping and are evaluated by the oncology and pathology departments.

### 2.5 The MYCN gene, the chromosome 1p36 and 11q analysis

MYCN gene status, chromosome 1p36 and 11q status were determined by FISH using standard methods. Tumors were considered amplified MYCN when copy number was increased > 5-fold.

### 2.6 Statistical analysis

Statistical analyzes were performed using SPSS software (version 28.0 for Windows; SPSS INC.). Continuous data were described as the mean ± standard deviation (mean ± SD) or median and interquartile, depending on whether they followed a normal distribution. The categorical variables are described as numbers. Differences between groups were compared using the Mann-Whitney U tests for the continuous variables, chi-squared tests, and Fisher’s exact test for the categorical variables. Receiver operating characteristic (ROC) curves were used to determine the best cut-off values of the level of NSE, LDH, LEGmax of the lesion and PET/CT metabolic parameters to predict the MYCN amplification and the positive of 1p36 and 11q. Binary logistic regression was used to analyze whether MYCN oncogene status, chromosome 11q and 1p36 statuses were influential factors leading to bone marrow infiltration and lymph node metastasis. Correlation analysis between MYCN deletions and chromosome 1p36,11q was performed using Spearman’s rank correlation analysis. Survival curves were created using the Kaplan-Meier method, and log-rank tests were used to determine whether the survival rates of the curves were statistically significant. All tests were two-sided, and a probability of less than 0.05 was considered statistically significant.

## 3 Results

### 3.1 Patients’ characteristics

We retrospectively studied 60 patients with 48 NB and 12 GNB (age < 18 years). This study included 34 males and 26 females with an average age of 34.78 months. The general characteristics of patients are summarized in **Table 1**. The status of the MYCN gene and chromosomes 1p36 and 11q was collected from 47 of 60 patients, of whom 34% were positive for MYCN amplification and approximately 38.3% and 36.2% for chromosome 1p36 and 11q deletions, respectively.

**Table 1:** General characteristics of 60 patients.

Characteristics		No. of patients	%
Age (months)	Median (range)	34 (2-144)	
Gender	Male	34	56.7
	Female	26	43.3
Histology	NB	48	80.0
	GNB	12	20.0
Location	Neck	1	1.7
	Chest	5	8.3
	Abdomen	53	88.3
	Pelvis	1	1.7
MYCN^#^	Amplified	16	34.0
	Not amplified	31	66.0
1P36^#^	Positive	18	38.3
	Negative	29	61.7
11q^#^	Positive	17	36.2
	Negative	30	63.8
BMB^##^	Positive	37	61.7
	Negative	23	38.3
Lymph node metastasis	Metastasis	50	83.3
	No metastasis	10	16.7
INRGSS	L1	7	11.7
	L2	9	15.0
	M	40	66.7
	Ms	4	6.7
INRG risk stratification^##^	Very low	5	9.8
	Low	5	9.8
	Intermediate	4	7.8
	High	37	72.5

^#^47 patients obtained the status of MYCN gene and chromosome 1p36 and 11q. ^##^51 patients obtained INRG risk stratification. BMB, bone marrow biopsy; INRGSS, International neuroblastoma risk group staging system; INRG, International neuroblastoma risk group.

### 3.2 Relationship between amplification of MYCN gene, deletion of chromosome 1p36 and 11q and clinicopathological features

The clinical biological characteristics and ^18^F-FDG PET/CT metabolic parameters of patients with the MYCN gene and chromosome 1p36 and 11q status are shown in **Table 2**. The differences in bone marrow involvement and lymph node metastases in patients with chromosome 11q deletion were statistically significant. Binary logistic regression analysis showed that chromosome 11q deletion was an independent factor affecting bone marrow involvement. The incidence of bone marrow involvement was 17.796 times higher in patients with chromosome 11q deletion than in those without chromosome 11q deletions, as shown in **Table 3**.

**Table 2:** General characteristics of MYCN gene and chromosomes 1p36 and 11q status.

	The status of the MYCN gene and chromosomes 1p36 and 11q								
	MYCN gene		p	1P36 chromosome		p	11q chromosome		p
	Amplification	Not amplification		mutation	No-mutation		mutation	No-mutation	
Age (months)									
≤18	4	6	0.606	3	7	0.570	2	8	0.240
>18	12	25		15	22		15	22	
Gender									
Male	7	18	0.351	6	19	0.032^#^	10	15	0.560
Female	9	13		12	10		7	15	
Histology									
NB	17	21	0.017^#^	18	20	0.064	15	23	1.000
GNB	0	9		1	8		3	6	
Primary site									
Chest	0	5	0.169	2	3	0.728	4	1	0.034^#^
Abdomen	16	25		16	25		12	29	
Pelvis	0	1		0	1		1	0	
INRG stage									
L1	0	5	0.066	0	5	0.123	1	4	0.230
L2	1	7		2	6		1	7	
M	14	19		16	17		15	18	
Ms	1	0		0	1		0	1	
INRG risk stratification									
Very low	0	5	0.013^#^	0	5	0.116	1	4	0.101
Low	0	5		1	4		0	5	
Intermediate	0	4		1	3		3	1	
High	16	16		16	16		13	19	
Bone marrow involvement									
Positive	8	22	0.156	9	21	0.120	16	14	0.01^#^
Negative	8	9		9	8		1	16	
Lymph node metastasis									
metastasis	14	26	1.000	16	24	0.692	17	23	0.039^#^
No metastasis	2	5		2	5		0	7	
LDH (IU/L)	2637.000±1334.187	547.807±223.195	<0.001^#^	1956.611±1356.043	826.034±1014.193	0.002^#^	895.823±727.453	1464.833±1465.428	0.142
NSE	521.150±150.790	249.639±170.358	<0.001^#^	415.967±211.561	296.200±195.438	0.054	371.565±179.180	325.353±223.825	0.470
LEGmax	10.975(8.685,13.083)	7.550(4.630,10.180)	0.050^#^	10.830(8.405,13.225)	7.320(4.920,10.080)	2.18	7.670(6.800,10.080)	9.575(5.738,12.550)	0.204
SUVmax	9.558±4.642	6.532±3.500	0.016^#^	7.681±3.544	7.489±4.525	0.879	6.384±2.458	8.230±4.748	0.144
SUVmean	4.194±1.490	2.953±1.571	0.012^#^	3.546±1.873	3.270±1.501	0.279	3.270±1.406	3.435±1.778	0.743
MTV	353.904±194.387	262.900±379.524	0.374	451.140±455.761	196.270±160.655	0.008^#^	223.506±185.535	333.759±384.738	0.724
TLG	1549.191±1051.693	617.908±683.038	<0.001^#^	1396.290±1213.845	648.586±550.403	0.006^#^	617.068±453.706	1115.069±1078.824	0.039^#^

NB, Neuroblastoma; GNB, Ganglioneuroblastoma; INRG, International neuroblastoma risk group; SUVmax, maximum standardized uptake value; SUVmean, average standard uptake value; MTV, metabolic tumor volume; TLG, total lesion glycolysis; LEGmax, the maximal length of the lesion; LDH, lactate dehydrogenase; NSE, neuron-specific enolase. #p<0.05.

**Table 3:** MYCN oncogene status, chromosome 11q and 1p36 status as factors influencing bone marrow infiltration.

Modalities	B	Exp(B)	p	95% lower	95% upper
MYCN	-0.040	0.961	0.963	0.175	5.273
1P36	-0.901	0.406	0.289	0.077	2.146
11q	2.879	17.796	0.011^#^	1.945	162.812

#p<0.05.

### 3.3 Relationship of different states of MYCN gene, chromosome 1p36 and 11q with PFS

**Figure 1:**
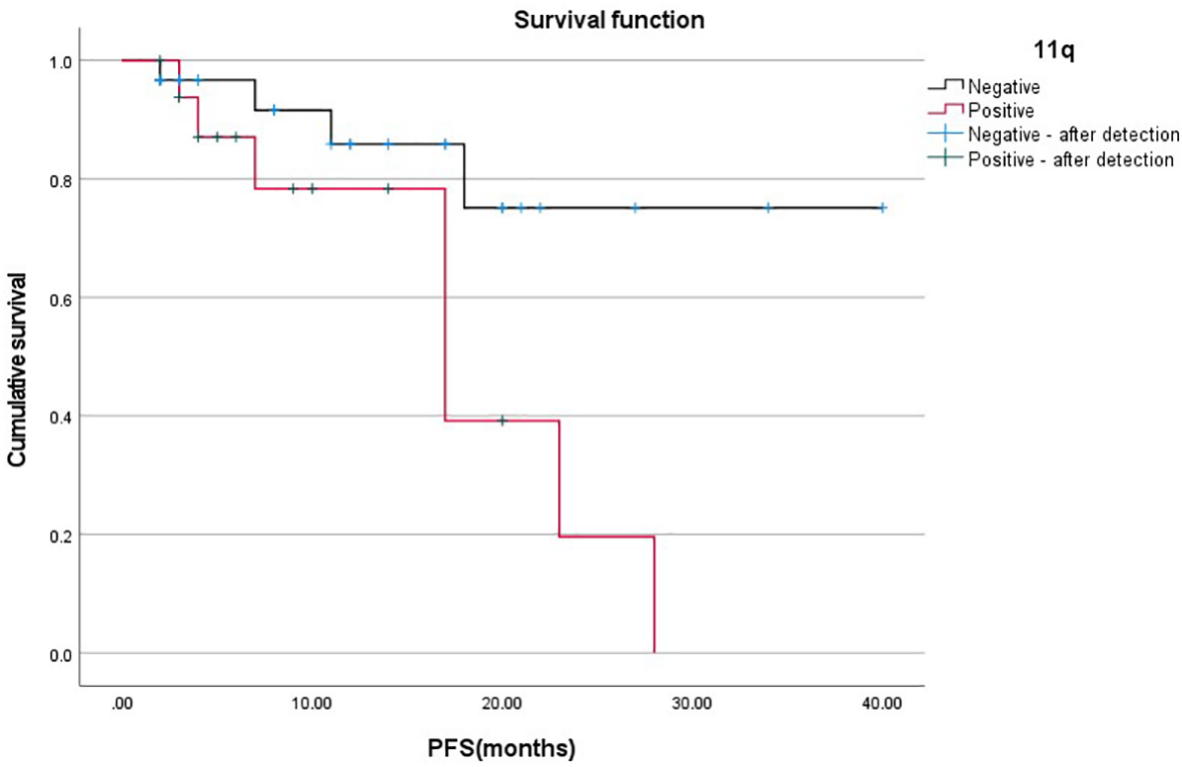
Relationship between 11q gene deletion and PFS.

The PFS between chromosome 11q deletion and chromosome 11q non-deletion group PFS is significantly different(X^2 =^ 5.314, p=0.021), as shown in **Figure 1**. The chromosome 11q non-deletion group has a longer PFS than the chromosome 11q deletion group. MYCN gene amplification and chromosome 1p36 deletion are not associated with PFS.

### 3.4 Correlation between MYCN gene amplification, chromosomes 1p36 and 11q deletion

Using Spearman’s rank correlation analysis, it can be concluded that MYCN gene amplification was positively correlated with chromosome 1P36 deletion (p=0.002, rs=0.450), while it was not correlated with chromosome 11q deletion (p=0.077, rs=-0.260) in **Table 4**. The deletion of chromosome 1P36 was not correlated with the deletion of chromosome 11q (p=0.356, rs=-0.138). The clinical biological characteristics and ^18^F-FDG PET/CT metabolic parameters of the different combinations with the different status of MYCN gene and chromosome 1p36 and 11q are shown in **Table 5**.

**Table 4:** Correlation between MYCN gene, chromosome 1p36 and 11q.

			MYCN		p	Rs
			Amplification	Not amplification		
1p36	Deletion		11	7	0.002	0.450
	No-deletion		5	24		
11q	Deletion		3	14	0.077	-0.260
	No-deletion		13	17		

**Table 5:** The clinical biological characteristics and ^18^F-FDG PET/CT metabolic parameters of the different combinations with the status of MYCN gene and t chromosomes 1p36 and 11q.

	MYCN gene amplification and chromosome 1P36 deletion	MYCN gene and chromosome 11q deletion	chromosome 1P36 and 11q deletion	MYCN gene amplification, chromosome 1P36 and 11q deletion
Total	10	2	4	1
Age (months)	1≤18 month; 9>18 month	108 month; 41 month	4 >18 month	54 month
Gender	4 male; 6 female	1 male; 1 female	1 male;2 female	female
Histology	10 neuroblastoma	2 neuroblastoma	3 neuroblastoma; 1 ganglioneuroblastoma	neuroblastoma
Primary site	10 Abdomen	2 Abdomen	2 Chest; 2 Abdomen	Abdomen
INRG stage	9 M; 1 L2	2 M	4 M	M
INRG risk stratification	10 High	2 High	4 High	High
Bone marrow involvement	2 Positive; 8 negative	2 Positive	4 Positive	Negative
Lymph node metastasis	8 Metastasis; 2 No metastasis	2 Metastasis	4 Metastasis	Metastasis
LDH	2686.5 (1680.0,3291.5)	1471; 1704	736.0 (551.75,1212.0)	1817
NSE	569.4 (462.0,600)	542; 600	200.5 (347.5,553.75)	53.60
LEGmax	11.635 (9.803,13.216)	7.32; 11.35	5.575 (4.035,10.758)	13.53
SUVmax	9.165 (6.798,10.743)	6.93; 9.13	5.575 (4.035,6.500)	13.47
SUVmean	4.400 (2.730,5.118)	4.17; 4.93	2.585 (1.540,3.645)	6.37
MTV	327.40 (228.455,616.450)	212.50; 349.79	252.055 (65.513,704.415)	492.74
TLG	1222.510 (910.298,3220.478)	898.75; 1647.60	260.625(115.578,1096.213)	3183.75
Follow-up status	7 No progression; 3 Progression (1 die)	2 No progression	2 No progression; 2 Progression	Progression (die)

LDH, lactate dehydrogenase; NSE, neuron-specific enolase; INRG, International neuroblastoma risk group; SUVmax, maximum standardized uptake value; SUVmean, average standard uptake value; MTV, metabolic tumor volume; TLG, total lesion glycolysis; LEGmax, the maximal length of the lesion.

### 3.5 Performance of ROC curves to predict MYCN amplification and chromosome 1p36 and 11q deletion

**Figure 2:**
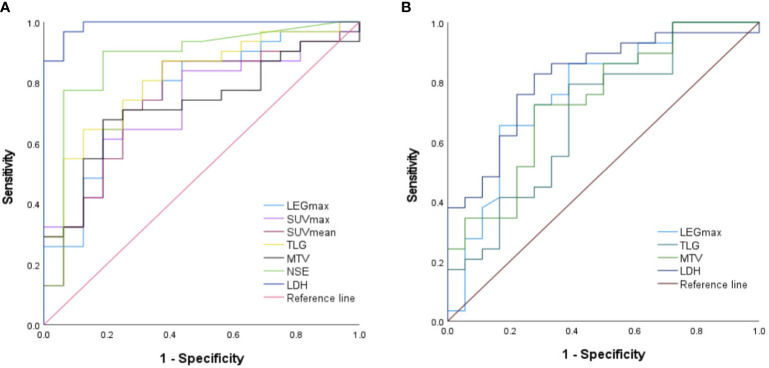
Performance of ROC curves predict the **(A)** MYCN gene amplification and **(B)** chromosome 1p36 deletion..

NSE and LDH levels, LEGmax, SUVmax, SUVmean, MTV, and TLG all predicted MYCN gene amplification (all p < 0.05), with LDH being the best, as shown in **Table 6** and **Figure 2A**. LDH levels, LEGmax and MTV, TLG all predicted the deletion of chromosome 1p36 (all p < 0.05), with the LDH being the best, while NSE, SUVmax and SUVmean did not (all p > 0.05), as shown in **Table 6** and **Figure 2B**. NSE and LDH levels, LEGmax, SUVmax, SUVmean, MTV and TLG could not predict chromosome11q deletion (all p>0.05).

**Table 6:** Performance of ROC curves predict the MYCN gene amplification and chromosome 1p36 deletion.

		MYCN gene							chromosome 1P36			
		NSE	LDH	LEGmax	SUVmax	SUVmean	MTV	TLG	LDH	LEGmax	MTV	TLG
p		0.001^#^	0.001^#^	0.003^#^	0.012^#^	0.007^#^	0.013^#^	0.001^#^	0.001^#^	0.002^#^	0.007^#^	0.034^#^
Optimal threshold		>472	>927	>8.75	>6.92	>4.14	>209.02	>562.41	>792	>7.99	>229.52	>982.43
AUC		0.875	0.990	0.764	0.726	0.742	0.724	0.813	0.814	0.767	0.736	0.686
Sensitivity		0.813	0.938	0.750	0.813	0.630	0.813	0.875	0.722	0.833	0.722	0.611
Specificity		0.903	0.968	0.710	0.613	0.871	0.677	0.645	0.828	0.655	0.724	0.793
95% CI	Upper limit	0.746	0.906	0.618	0.576	0.594	0.574	0.687	0.689	0.621	0.588	0.525
	Lower limit	0.953	1.000	0.876	0.846	0.858	0.844	0.938	0.939	0.914	0.883	0.847

NSE, neuron-specific enolase; LDH, lactate dehydrogenase; SUVmax, maximum standardized uptake value; SUVmean, average standard uptake value; MTV, metabolic tumor volume; TLG, total lesion glycolysis; LEGmax, the maximal length of the lesion. #p<0.05.

### 3.6 Relationship of LDH and NSE levels, age, gender, ^18^F-FDG PET/CT metabolic parameters with PFS

In addition to gender (p=0.543), the age, the levels of LDH and NSE, the LEGmax, ^18^F-FDG PET/CT metabolic parameters (all p < 0.01), including SUVmax, SUVmean, MTV and TLG, were all associated with PFS, as shown in **Table 7**.

**Table 7:** Relationship of the age, gender and the levels of LDH, NSE and ^18^F-FDG PET/CT metabolic parameters with PFS.

	Age	Gender	LDH	NSE	LEGmax	SUVmax	SUVmean	MTV	TLG
*χ* ^2^	113.907	0.369	154.810	100.147	154.802	154.832	152.396	154.832	154.832
p	<0.01^#^	0.543	<0.01^#^	<0.01^#^	<0.01^#^	<0.01^#^	<0.01^#^	<0.01^#^	<0.01^#^

LDH, lactate dehydrogenase; NSE, neuron-specific enolase; SUVmax, maximum standardized uptake value; SUVmean, average standard uptake value; MTV, metabolic tumor volume; TLG, total lesion glycolysis; LEGmax, the maximal length of the lesion. #p<0.05.

## 4 Discussion

^123^I-MIBG scintigraphy and ^68^Ga-1,4,7,10-tetraazacyclododecane1,4,7,10-tetraacetic acid (DOTA) peptides are now the mainstay nuclear imaging agents for neuroblastoma (22). ^123^I-MIBG imaging is primarily due to its structural similarity to norepinephrine, tissue that selectively concentrates ^123^I-MIBG with abundant adrenergic innervation, and mostly neuroectodermal tissues, including neuroblastoma (23). High affinity binding of different ^68^Ga-DOTA peptides to SSTR for PET/CT imaging (24, 25). Somatostatin receptors (SSTR) are expressed in 77%–89% of neuroblastoma cells. Most studies have confirmed the feasibility of ^18^F-FDG PET for neuroblastoma imaging, which is seen as an important and widespread alternative to ^123^I-MIBG scanning, especially for non-MIBG uptake (26). ^18^F-FDG scan may be useful in assessing the full extent of disease involvement, particularly at therapeutic decision points (26). We report the ^18^F-FDG PET/CT metabolic parameters in newly diagnosed pediatric neuroblastoma and their association with the other indicators of disease clinical biological characteristics and survival status.

In our study, 47 of 60 patients had molecular pathology results, including the status of MYCN gene and chromosomal 1p36 and 11q. We found that the rates of MYCN gene amplification, the chromosome 1p36 and 11q deletion were 34%, 38.3% and 36.2%, respectively, which is in general agreement with other reports on pediatric neuroblastoma (6, 10, 19, 27). In our study, we also found a correlation between MYCN gene amplification and chromosome 1p36 deletions (27, 28). In contrast, there was no correlation between MYCN gene amplification and chromosome 11q deletion and between chromosome 1p36 and 11q deletions.

Much research has found that MYCN gene amplification, chromosome 1p36 and 11q deletions can predict stage and prognosis in patients with neuroblastoma. In our study, MYCN gene amplification and chromosome 11q deletion predict high-risk stratification. What’s more, chromosomal deletion in 11q is highly correlated with lymph node metastasis and bone marrow involvement, suggesting a causal relationship between this region and the disease stage. Based on the Kaplan Meier method and log-rank test, chromosome11q deletion can affect PFS, and patients with chromosome 11q deletions are more likely to progress or die. These results are consistent with other reports of chromosome 11q mutations in pediatric neuroblastoma (27, 28). In addition, we also found that MYCN oncogene amplification is only found in patients with NB, about 100%.

^18^F-FDG PET/CT can be used for tumor characterization and prognostic assessment in patients with neuroblastoma (18). However, fewer reports have been reported on the ability of ^18^F-FDG PET/CT metabolic parameters in predicting the case of MYCN oncogene amplification, chromosome 1p36 and 11q deletion. In our study, the levels of LDH, NSE and LEGmax, SUVmax, SUVmean, TLG were higher in the MYCN gene amplification group than in the non-amplified group, and the differences were statistically significant in both groups, except for MTV. Meanwhile, the levels of LDH, the lesion MTV and TLG were higher in the chromosome 1p36 deletion group than in the non-deletion group. The differences were statistically significant in both groups. The lesion MTV was higher in the chromosome 11q deletion group than in the non-deletion group, and the difference between them was statistically significant.

Based on receiver operating characteristic (ROC) curve analysis, we obtained optimal threshold values of SUVmax 6.92, SUVmean 4.14, MTV 209.02 cm^3^, TLG 562.41, NSE 472.0 ng/ml, LDH 927.0 U/L and LEGmax 8.75cm, which can determine whether amplification of the MYCN gene occurred. Meanwhile, we also obtained optimal threshold values of MTV 229.52 cm^3^, TLG 982.43, LDH 792 U/L and LEGmax 7.99cm, these can determine whether a deletion of chromosome 1p36 has occurred. Surprisingly, there are no ^18^F-FDG PET/CT metabolic parameters and laboratory indicators that can effectively determine whether chromosome 11q is mutated.

In addition to gender (p=0.543), the age, the levels of LDH and NSE, the LEGmax, ^18^F-FDG PET/CT metabolic parameters (all p < 0.01), including SUVmax, SUVmean, MTV and TLG are all associated with PFS. In ^18^F-FDG PET/CT metabolic parameters, higher SUVmax, SUVmean, MTV and TLG were associated with worse PFS.

There are several limitations to our study. First, our results are inherently subject to selection bias as a retrospective study. Second, it may be that the small number of patients collected does not allow for a valid assessment of the differences in metabolic parameters of ^18^F-FDG PET/CT in multiple patients with simultaneous several molecular deletions. Third, we only performed correlation analyzes for PFS because of the short follow-up of patients (2-42 months; median time 9.5 months). It is also possible that the observation of the influence of the MYCN oncogene amplification and the chromosome 1p36 deletions on PFS was limited by the short follow-up period. Another shortcoming is that the status of chromosome 17q was not evaluated in our hospital.

## 5 Conclusion

The LDH levels, LEGmax, MTV and TLG can effectively predict the status of the MYCN oncogene and chromosome 1p36 in pediatric neuroblastoma and ganglioneuroblastoma. Those patients with chromosome 11q deletions are more likely to develop bone marrow involvement and lymph node metastases, showing a worse progression-free survival.

## Data availability statement

The raw data supporting the conclusions of this article will be made available by the authors, without undue reservation.

## Ethics statement

The studies involving human participants were reviewed and approved by Ethics Committee of Cancer Hospital Affiliated to Shandong First Medical University. Written informed consent to participate in this study was provided by the participants’ legal guardian/next of kin. Written informed consent was obtained from the minor(s)’ legal guardian/next of kin for the publication of any potentially identifiable images or data included in this article.

## Author contributions

JR: acquisition of data, analysis of data, drafting of the manuscript. YZ: analysis of data and revision of the manuscript. ZF: revision of the manuscript, supervision. All authors contributed to the article and approved the submitted version.
